# CHD7 promotes neural progenitor differentiation in embryonic stem cells via altered chromatin accessibility and nascent gene expression

**DOI:** 10.1038/s41598-020-74537-4

**Published:** 2020-10-15

**Authors:** Hui Yao, Douglas F. Hannum, Yiwen Zhai, Sophie F. Hill, Ricardo D.’Oliveira Albanus, Wenjia Lou, Jennifer M. Skidmore, Gilson Sanchez, Alina Saiakhova, Stephanie L. Bielas, Peter Scacheri, Mats Ljungman, Stephen C. J. Parker, Donna M. Martin

**Affiliations:** 1 Department of Pediatrics, University of Michigan, 8220C MSRB III, 1150 W. Medical Center Dr., Ann Arbor, MI 48109-5652 USA; 2 Department of Biostatistics, University of Michigan, Ann Arbor, MI USA; 3 Center of Genetic and Prenatal Diagnosis, The First Affiliated Hospital of Zhengzhou University, Zhengzhou, China; 4 College of Literature, Science, and the Arts, University of Michigan, Ann Arbor, MI USA; 5 Department of Computational Medicine and Bioinformatics, University of Michigan, Ann Arbor, MI USA; 6 Department of Genetics and Genome Sciences, Case Western Reserve University, Cleveland, OH USA; 7 Department of Human Genetics, University of Michigan, Ann Arbor, MI USA; 8 Department of Radiation Oncology, University of Michigan, Ann Arbor, MI USA

**Keywords:** Development, Epigenetics in the nervous system

## Abstract

CHARGE syndrome, a rare multiple congenital anomaly condition, is caused by haploinsufficiency of the chromatin remodeling protein gene *CHD7* (Chromodomain helicase DNA binding protein 7). Brain abnormalities and intellectual disability are commonly observed in individuals with CHARGE, and neuronal differentiation is reduced in CHARGE patient-derived iPSCs and conditional knockout mouse brains. However, the mechanisms of CHD7 function in nervous system development are not well understood. In this study, we asked whether CHD7 promotes gene transcription in neural progenitor cells via changes in chromatin accessibility. We used *Chd7* null embryonic stem cells (ESCs) derived from *Chd7* mutant mouse blastocysts as a tool to investigate roles of CHD7 in neuronal and glial differentiation. Loss of *Chd7* significantly reduced neuronal and glial differentiation. Sholl analysis showed that loss of *Chd7* impaired neuronal complexity and neurite length in differentiated neurons. Genome-wide studies demonstrated that loss of *Chd7* leads to modified chromatin accessibility (ATAC-seq) and differential nascent expression (Bru-Seq) of neural-specific genes. These results suggest that CHD7 acts preferentially to alter chromatin accessibility of key genes during the transition of NPCs to neurons to promote differentiation. Our results form a basis for understanding the cell stage-specific roles for CHD7-mediated chromatin remodeling during cell lineage acquisition.

## Introduction

CHARGE syndrome is a multisystem developmental disorder characterized by *C*oloboma, *H*eart defects, *A*tresia of the choanae, *R*etardation of growth and development, *G*enital hypoplasia, and *E*ar malformations including deafness and vestibular disorders^1^. Nervous system abnormalities are also common among individuals with CHARGE, including brain malformations (i.e., hindbrain anomalies, brainstem anomalies, and ventricular enlargement), abnormal sensory organ structure and function (i.e., ocular coloboma and sensorineural hearing loss), and impaired cranial nerves (hypoplasia or aberrant innervation)^2–5^. Together, these malformations contribute to a wide range of cognitive defects and behavioral challenges for individuals with CHARGE, including learning disabilities, speech and communication difficulties, motor incoordination, and autistic behaviors^6,7^.

Pathogenic variants in *CHD7* are a major diagnostic criterion for CHARGE syndrome and are present in over 71% of cases^8^. *CHD7* encodes *C*hromodomain *H*elicase *D*NA binding protein 7 (CHD7), an ATP-dependent chromatin remodeling enzyme and member of the Group III CHD family of chromatin remodelers^9^. *Chd7* is expressed highly in the adult mouse subventricular zone (SVZ) and subgranular zone (SGZ) where neural progenitors contribute to formation of adult born neurons^10^. CHD7 has been broadly implicated in neurogenesis in multiple areas of the nervous system, during development and beyond. In mice, heterozygous loss of *Chd7* mimics human CHARGE syndrome, with reduced volume of the brain, cortex, and cerebellum compared to controls^3^. In adult *Chd7* conditional knockout mice, SVZ neural stem/progenitor cells exhibit impaired neurogenesis^11^, and loss of *Chd7* leads to reduced neurogenesis and abnormal dendritic development of newly born neurons in the adult mouse SVZ and SGZ^10^. Adult hippocampal loss of *Chd7* causes premature neuron production and depletion of neural stem cells^12^. In the telencephalon, midbrain and spinal cord, neuroepithelial thickness is reduced in E10.5 heterozygous gene trap (*Chd7*^*Gt/*+^) embryos^13^. Together, these observations implicate CHD7 in a wide variety of central nervous system and neuron-specific processes.

In addition to central nervous system defects, *Chd7* deficiency has been implicated in peripheral nervous system neurogenesis, including in the olfactory and auditory epithelia^14,15^. In the ear, germline and conditional loss of *Chd7* leads to a severe reduction in neuroblasts in the developing otic epithelium and the cochleovestibular ganglion^15^. Taken together, these observations demonstrate that CHD7 promotes neurogenesis and gliogenesis in early embryonic central and peripheral nervous system development.

CHD7, like other chromatin remodelers, is known to regulate chromatin accessibility using the energy of ATP to reposition nucleosomes^16,17^. CHD7 is required in cerebellar granule neural progenitor cells for maintenance of open chromatin differentiation^18^, regulation of neuronal differentiation^10,15^, and fate determination by influencing enhancer activity^18,19^. In mouse embryonic stem cells (ESCs), CHD7 co-localizes with transcription factors including OCT4, SOX2, and NANOG at active enhancers to promote expression of pluripotency markers in ESC-specific genes^19^. In addition, CHD7 is a reader of H3K4me1, a canonical histone mark of enhancers, and its binding pattern varies with developmental stage from ESCs to neural progenitor cells (NPCs)^19,20^. CHD7 dosage reduction leads to a fate switch from neural epithelial to neural crest cell lineages in induced pluripotent stem cells (iPSCs) derived from individuals with CHARGE and *CHD7* pathogenic variants^21^. CHD7 also regulates central nervous system lineages in patient derived iPSCs via SOX21 and BRN2 activation at super enhancers^22^.Together, these observations suggest that CHD7 regulates enhancer activity and expression of nervous system genes in iPSCs. However, the temporal and genome-region specific requirements for CHD7 in developing neurons.

To better understand CHD7 function in mammalian neurogenesis, we derived *Chd7* null ESCs from *Chd7* mutant blastocysts. Neuronal lineage differentiation was induced in vitro and the effects of CHD7 on gene expression (using Bru-Seq) and chromatin accessibility (using ATAC-seq) were analyzed. We found that loss of *Chd7* in ESCs leads to decreased neuronal and glial cell differentiation and impaired neuronal complexity and neurite length in differentiated neurons. We also observed modified chromatin accessibility and altered expression of neural-specific genes with loss of *Chd7*. Our results suggest developmental stage-specific roles for CHD7 in neuronal development via changes in chromatin accessibility and gene transcription.

## Results

### Loss of *Chd7* does not disrupt development or proliferation of ESCs and NPCs

**Figure 1:**
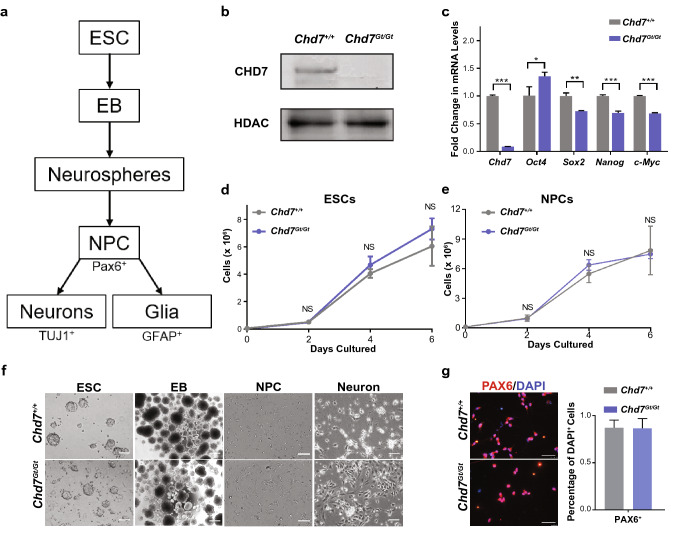
CHD7 promotes differentiation of neurons from ESCs. (**a**) Flow chart of ESC differentiation over 14 days into primary and secondary EBs, neurospheres, NPCs, neurons and glia. The protocol involves sequential treatment of ESCs with specified media. After secondary EB formation, EBs are cultured in suspension to form neurospheres to generate NPC cell lines or plated on Poly-l-lysine coated coverslips for direct induction into PAX6^+^ NPCs and subsequent differentiation into TUJ1^+^ neurons or GFAP^+^ glia. (**b**) Western blot showing absence of CHD7 protein in *Chd7*^*Gt/Gt*^ ESCs. (**c**) ESC pluripotency genes are expressed based on RT-qPCR of steady state RNA in both *Chd7*^+*/*+^ and *Chd7*^*Gt/Gt*^ ESCs (N = 3). Significant differences were found in all of the genes. *P < 0.05, **P < 0.01, ***P < 0.001. (**d**) *Chd7*^*Gt/*Gt^ and *Chd7*^+*/*+^ ESCs grow at similar rates over 6 days in culture (N = 3 for each time point). (**e**) *Chd7*^+*/*+^ and *Chd7*^*Gt/Gt*^ NPCs grow at similar rates over 6 days in culture (N = 3 for each time point). (**f**) *Chd7*^+*/*+^ and *Chd7*^*Gt/Gt*^ ESCs, embryoid bodies (EBs) and NPCs exhibit similar morphologies. Scale bars = 50 µm. (**g**) Immunostaining for PAX6 (left) in NPCs derived from *Chd7*^+*/*+^ and *Chd7*^*Gt/Gt*^ ESCs. Quantification of immunostaining is shown on the right. NPCs show no difference between *Chd7*^+*/*+^ and *Chd7*^*Gt/Gt*^ in the proportion of PAX6^+^ cells (P = 0.94). Data of three independent experiments are represented as mean ± SEM. Unpaired student’s *t-*test was used for statistical analysis. Scale bars = 50 µm.

**Figure 2:**
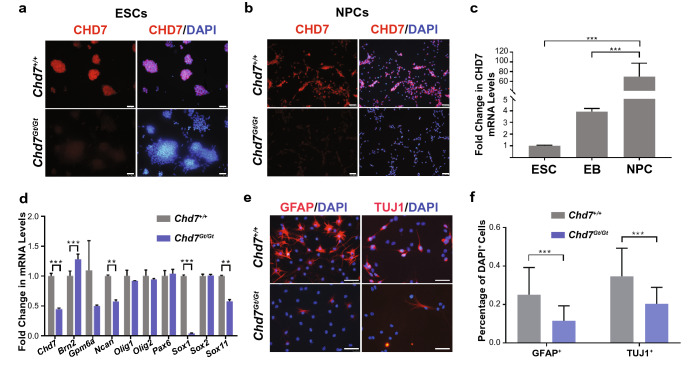
CHD7 promotes neuronal and glial differentiation in established NPC lines. (**a**,**b**) Immunostaining identifies CHD7 in *Chd7*^+*/*+^ but not in *Chd7*^*Gt/Gt*^ ESCs (a) or NPCs. (**b**). Scale bars = 50 µm. (**c**) *Chd7* mRNA levels significantly increase as *Chd7*^+*/*+^ cells transition from ESCs to EBs and NPCs (N = 3). *Chd7* mRNA levels also increase significantly between *Chd7*^+*/*+^ ESCs and NPCs (68.68 ± 13.08, P = 0.006) and between *Chd7*^+*/*+^ EBs and NPCs (65.77 ± 13.08, P = 0.007). ***P < 0.001. (**d**) RT-qPCR of neural progenitor genes in *Chd7*^*Gt/Gt*^ vs *Chd7*^+*/*+^ ESC-derived NPCs. (**e**) Immunostaining for the glial marker GFAP in cells derived from *Chd7*^+*/*+^ and *Chd7*^*Gt/Gt*^ NPCs, and for the neuronal marker TUJ1 in cells derived from *Chd7*^+*/*+^ and *Chd7*^*Gt/Gt*^ NPCs. Scale bars = 50 µm. (**f**) Quantification of the immunostaining in (**e**). There is a significant reduction in the proportion of GFAP^+^ (− 0.14 ± 0.02, P = 5.8 × 10^–9^) and TUJ1^+^ cells (− 0.14 ± 0.04, P = 9.7 × 10^–4^) in *Chd7*^*Gt/Gt*^ compared to *Chd7*^+*/*+^. Unpaired student’s *t-*test was used for statistical analysis. ***P < 0.001.

*Chd7* is highly expressed in developing neurons in vivo^14^; thus, we hypothesized that CHD7 may play an active role in the differentiation of embryonic stem cells (ESCs) to neural progenitor cells (NPCs) and subsequently to neurons and glia (Fig. 1a). To test this hypothesis, we generated ESCs from mouse *Chd7*^+*/*+^ and *Chd7*^*Gt/Gt*^ blastocysts. Western-blotting (Fig. 1b, Supplementary Fig. 1) and immunostaining (Fig. 2a) showed an absence of CHD7 protein, and qRT-PCR showed significantly reduced *Chd7* mRNA (Fig. 1c), in *Chd7*^*Gt/Gt*^ vs. *Chd7*^+*/*+^ ESCs. These observations validate prior studies showing that the *Chd7*^*Gt*^ allele gives rise to a β-gal-*Chd7* fusion transcript but no detectable CHD7 protein, confirming the null status of *Chd7*^*Gt/Gt*^ ESCs^13^.

As a first step toward characterizing *Chd7* null ESCs, we measured expression of the pluripotency markers *Oct4*, *Sox2*, *Nanog*, and *c-Myc*. All markers showed modest, albeit significant, differences in expression in *Chd7*^*Gt/Gt*^ ESCs compared to *Chd7*^+*/*+^ cells (P < 0.05), with fold changes ranging from 0.69 and 1.35 (Fig. 1c). Neither *Chd7*^+*/*+^ or *Chd7*^*Gt/Gt*^ ESCs showed defects in proliferation at passages up to P21 when measured over 6 days in standard ESC culture medium (Fig. 1d). In addition, there were very few (~ 2%) apoptotic cells as identified by immunostaining for cleaved Caspase 3 in *Chd7*^+*/*+^ and *Chd7*^*Gt/Gt*^ ESCs (Supplementary Fig. 2a,b), suggesting that *Chd7* deficiency does not alter ESC apoptosis. Moreover, no obvious differences in morphology were observed between *Chd7*^+*/*+^ and *Chd7*^*Gt/Gt*^ ESCs (Fig. 1f). These data indicate that loss of *Chd7* does not disrupt ESC morphology, proliferation, or cell death, but may have mild effects on pluripotency markers, which is consistent with other observations^19^.

ESCs were differentiated into NPCs via directed differentiation without Leukemia Inhibitory Factor (LIF) (Fig. 1f). *Chd7*^+*/*+^ and *Chd7*^*Gt/Gt*^ ESCs were successfully differentiated to embryoid bodies (EBs) and NPCs, indicating that CHD7 is not essential for differentiation to neuroectoderm and progenitor fates. The percentage of *Chd7*^+*/*+^ and *Chd7*^*Gt/Gt*^ NPCs expressing the marker PAX6 were similar (Fig. 1g), indicating that CHD7 is not necessary for the transition from ESCs to NPCs. Other NPC markers showed similar results (Supplementary Fig. 3). Over a 6-day period, *Chd7*^+*/*+^ and *Chd7*^*Gt/Gt*^ NPC cell counts were comparable (Fig. 1e), suggesting no major defects in cell proliferation or survival with *Chd7* loss. There were also no differences in the number of apoptotic NPCs (based on Caspase 3 immunostaining) in *Chd7*^+*/*+^ vs. *Chd7*^*Gt/Gt*^ NPCs (Supplementary Fig. 2c,d). These observations demonstrate that CHD7 is dispensable for the generation and survival of NPCs from ESCs.

### CHD7 is required for differentiation of NPC into neurons and glia

CHD7 is expressed at the protein and mRNA levels in *Chd7*^+*/*+^ NPCs, as confirmed by immunostaining (Fig. 2b) and qRT-PCR (Fig. 2d). As expected, CHD7 protein was absent in *Chd7*^*Gt/Gt*^ NPCs (Fig. 2b) and *Chd7* mRNA (reflecting a chimeric β-gal-*Chd7* transcript from the *Chd7*^*Gt*^ allele) was reduced in *Chd7*^*Gt/Gt*^ NPCs (Fig. 2d). Interestingly, *Chd7* mRNA levels gradually increased during neural differentiation of *Chd7*^+*/*+^ ESCs to EBs and NPCs (Fig. 2c). Expression of multipotency factors *Brn2, Ncan, Sox1,* and *Sox11* was significantly different between *Chd7*^+*/*+^ and *Chd7*^*Gt/Gt*^ NPCs (P < 0.05) (Fig. 2d). Notably, *Sox1* mRNA levels were reduced to 4% of wild type levels in *Chd7*^*Gt/Gt*^ NPCs (P = 1.1 × 10^–6^), consistent with previous studies showing that *Sox1* promotes progenitor cell cycle exit and establishment of neuroectodermal lineages in mouse cells^23^.

Neurons and glia arise from common progenitors, with neurogenesis preceding gliogenesis during development^24^. In order to test the function of *Chd7* in NPC transition to neural and glial lineages, we established NPC cell lines from *Chd7*^+*/*+^ and *Chd7*^*Gt/Gt*^ ESCs. There were no differences in the proportion of *Chd7*^+*/*+^ and *Chd7*^*Gt/Gt*^ cells expressing the neural markers NEUN, MAP2, Calretinin, or Neurofilament-200 (Supplementary Fig. 4). However, the proportion of TUJ1^+^ cells decreased from 24% in *Chd7*^+*/*+^ to 10% in *Chd7*^*Gt/Gt*^ (SD = 4%, P = 9.7 × 10^–4^) (Fig. 2e,f). The proportion of GFAP^+^ cells was also reduced from 34% in *Chd7*^+*/*+^ to 20% in *Chd7*^*Gt/Gt*^ (SD = 2%, P = 5.8 × 10^–9^) (Fig. 2e,f). These results suggest that CHD7 promotes NPC differentiation into both glial and neuronal lineages in mouse ESC-derived NPCs, instead of selectively regulating one lineage at the expense of the other.

### Loss of Chd7 impairs the number, length and complexity of neurites in NPC-derived neurons

**Figure 3:**
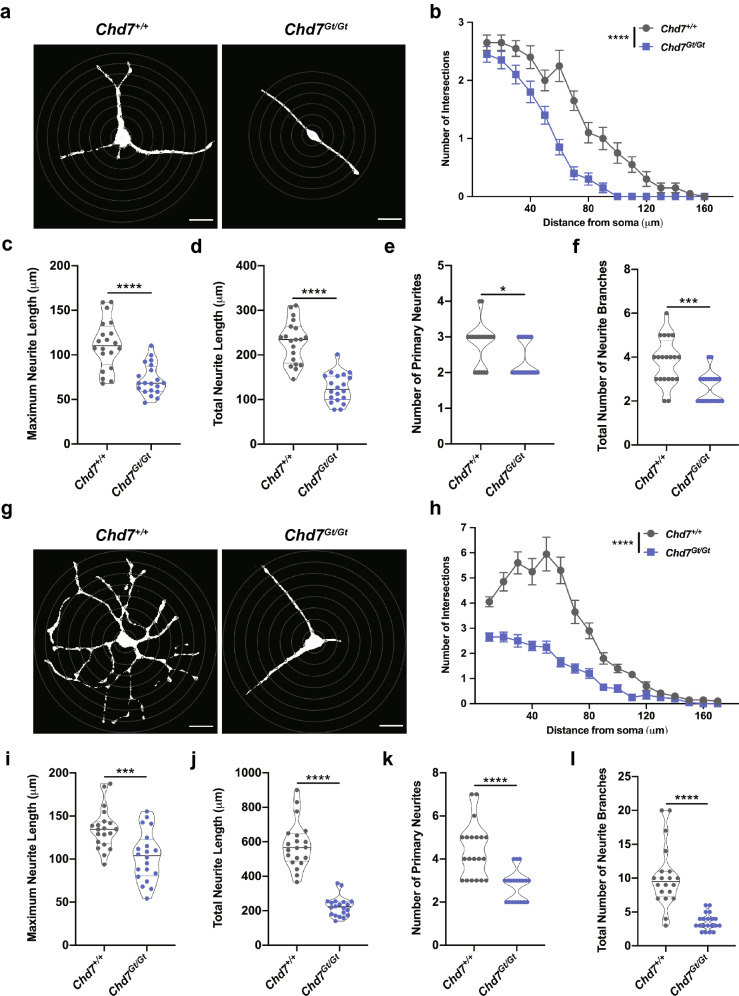
Loss of *Chd7* impairs the number, length, and complexity of neurites in NPC-derived neurons. (**a**–**f**) Neurons at DIV5. (**g**–**l**) Neurons at DIV9. (**a**) Representative images of *Chd7*^+*/*+^ neurons (left) and *Chd7*^*Gt/Gt*^ neurons (right). For Sholl analysis, the total number of neurite crossings was counted at each circle with a radius increasing in increments of 10 μm. Scale bar = 20 μm. (**b**) Sholl analysis of the neuritic complexity of MAP2^+^ neurons. The data are shown as mean ± SEM (N = 20 per genotype). Two-way ANOVA was used for data analysis. ****P < 0.0001. (**c**,**d**) Loss of *Chd7* results in a significant decrease in the maximum and total neurite length of differentiated neurons. Each symbol represents a single MAP2^+^ neuron (N = 20 per genotype). Unpaired student’s *t-*test was used for statistical analysis. ****P < 0.0001. (**e**,**f**) Loss of *Chd7* causes a significant reduction in the number of primary neurites from the soma and total number of neurite branches of differentiated neurons. Each symbol represents a single MAP2^+^ neuron (N = 20 per genotype). Unpaired student’s *t-*test was used for statistical analysis. *P < 0.05; ***P < 0.001. (**g**) Representative images of *Chd7*^+*/*+^ neurons (left) and *Chd7*^*Gt/Gt*^ neurons (right) at DIV9. Radius increments are 10 μm for Sholl analysis, Scale bar = 20 μm. (**h**) Sholl analysis of MAP2^+^ neurons. The data are shown as mean ± SEM (N = 20 per genotype). ****P < 0.0001 by two-way ANOVA. (**i**,**j**) The maximum and total neurite length of differentiated neurons were significantly reduced in *Chd7*^*Gt/Gt*^ neurons compared to *Chd7*^+*/*+^ neurons. Each symbol represents a single MAP2^+^ neuron (N = 20 per genotype). Unpaired student’s *t-*test was used for statistical analysis. ***P < 0.001; ****P < 0.0001. (**k**,**l**) The number of primary neurites from the soma and total number of neurite branches were decreased in *Chd7*^*Gt/Gt*^ neurons compared to *Chd7*^+*/*+^ neurons. Each symbol represents a single MAP2^+^ neuron (N = 20 per genotype). Unpaired student’s *t-*test was used for statistical analysis. ****P < 0.0001.

Reduced percentages of cells expressing the neural marker TUJ1 and the glial marker GFAP in *Chd7*^*Gt/Gt*^ cultures suggests that CHD7 has broad functions in ESC-derived cell differentiation. To address this, we assayed the morphology of ESC-derived neurites using Sholl analysis on MAP2-positive cultures. Use of MAP2 instead of TUJ1 allowed us to label proportionately similar numbers of cells in cultures from both genotypes. Typical tracings of MAP2-positive neurons from DIV5 *Chd7*^+*/*+^ and *Chd7*^*Gt/Gt*^ neurons are shown in Fig. 3a. Sholl analysis of neuritic complexity shows a reduction in the number of intersections for neurites in *Chd7*^*Gt/Gt*^ compared to *Chd7*^+*/*+^ (Fig. 3b)*.* The average individual neurite length at DIV5 was significantly reduced from 111.8 (± 27.1) μm in *Chd7*^+*/*+^ neurons to 72.2 (± 16.8) μm in *Chd7*^*Gt/Gt*^ neurons (Fig. 3c). Total neurite length was also reduced from 229.4 (± 46.2) μm in *Chd7*^+*/*+^ neurons to 126.2 (± 37.8) μm in *Chd7*^*Gt/Gt*^ neurons (Fig. 3d). The average number of primary neurites extending from the soma was reduced from 2.8 (± 0.6) in *Chd7*^+*/*+^ neurons to 2.3 (± 0.5) in *Chd7*^*Gt/Gt*^ neurons (Fig. 3e). The total number of neurite branches was also reduced from 3.8 (± 1.1) in *Chd7*^+*/*+^ neurons to 2.6 (± 0.6) in *Chd7*^*Gt/Gt*^ neurons (Fig. 3f). Together, these data show that CHD7 is necessary for normal neurite length, complexity, and branching in ESC-derived neurons.

**Figure 4:**
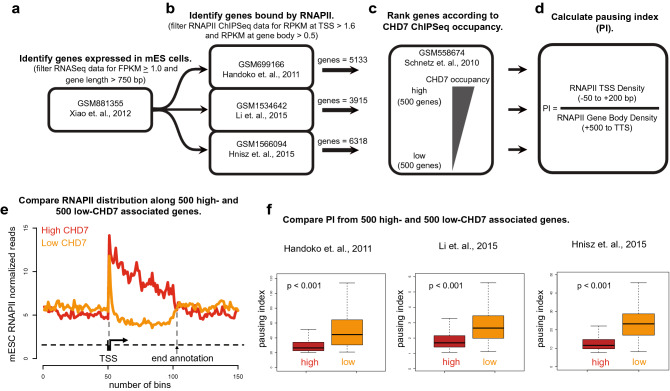
Genes with high CHD7 occupancy display low RNAPII pausing and increased RNAPII throughout the gene body. (**a**–**d**) Pipeline used to identify expressed genes occupied by RNAPII and CHD7 in mouse ESCs. (**e**) Meta-analysis profile of RNAPII occupancy along gene territories in ESCs as determined from previously published RNAPII ChIP-Seq data. Normalized RNAPII binding averages in 150 bins are shown to illustrate the general RNAPII distribution along genes with high (red) and low (orange) CHD7 occupancy as determined from ChIP-seq. (**f**) Boxplots comparing RNAPII promoter pausing indices from the three different RNAPII ChIP-Seq datasets in relationship to two CHD7-associated gene sets (high vs low). P value was determined using two-sample Wilcoxon test.

To further assess the role of *Chd7* in neuronal differentiation, we extended the culture time to DIV9. Typical tracings of MAP2-positive *Chd7*^+*/*+^ and *Chd7*^*Gt/Gt*^ neurons are shown in Fig. 4g. We observed similar and more severe impairments in neurite number, branching, and complexity at DIV9 compared to DIV5. Sholl analysis of neuritic complexity shows a reduction in the number of intersections for *Chd7*^*Gt/Gt*^ compared to *Chd7*^+*/*+^ (Fig. 3h)*.*The average maximum neurite length was reduced from 135.1 (± 23.2) μm in *Chd7*^+*/*+^ neurons to 103.1 (± 28.2) μm in *Chd7*^*Gt/Gt*^ neurons (Fig. 3i). Total neurite length was reduced from 581.5 (± 132.3) μm in *Chd7*^+*/*+^ neurons to 224.1 (± 55.6) μm in *Chd7*^*Gt/Gt*^ neurons (Fig. 3j). Additionally, the number of primary neurites extending from the soma ranged from 2 to 4 in *Chd7*^*Gt/Gt*^ neurons and from 3 to 7 in *Chd7*^+*/*+^ neurons (Fig. 3k). The total number of neurite branches was reduced on average from 10.2 (± 4.4) in *Chd7*^+*/*+^ neurons to 3.55 (± 1.2) in *Chd7*^*Gt/Gt*^ neurons (Fig. 3l). Taken together, these results suggest that *Chd7* is required over a nine-day period for multiple aspects of neuronal morphogenesis including neurite extension, branching, and complexity.

### CHD7 is enriched at genes with high RNA polymerase occupancy

The Drosophila orthologue of *Chd7*, *kismet*, has also been shown to positively regulate neural differentiation and promote transcriptional elongation on polytene chromosomes^25^. Given these observations, we asked whether mammalian CHD7 may also affect rates of transcriptional elongation, in addition to its known roles in activation or repression of target genes. A key control point for transcriptional elongation is RNA polymerase II (RNAPII) release from promoter-proximal pause sites^26^, whereby RNAPII pausing generates stable DNA secondary structures upon unwinding of double-stranded DNA. In mouse cerebellar granule cells, CHD7 interacts with Topoisomerase to facilitate chromatin unwinding^18^. Thus, CHD7 could regulate RNAPII pause release to help resolve DNA structures that stall transcriptional elongation.

To determine whether CHD7 localization correlates with RNAPII occupancy and gene expression, we accessed publicly available ChIP-Seq data for CHD7 and RNAPII as well as RNA-seq data, all derived from mouse ESCs. We used these data to calculate pausing index, defined as the ratio of Reads Per Kilobase of transcript, per Million mapped reads (RPKM) at the transcription start site (TSS), to RPKM at the gene body, for genes with high vs low CHD7 occupancy over the whole gene (Fig. 4). Mouse ESC RNA-Seq data were obtained from GEO dataset GSM881355 (Fig. 4a). From these data, we selected genes expressed with FPKM ≥ 1.0 and length > 750 bp. Next, genes bound by RNAPII were identified by analysis of three RNAPII ChIP-Seq data sets (GSM699166, GSM1534642, GSM1566094) for RPKM at TSS > 1.6 and RPKM at the gene body > 0.5 (Fig. 4b). Genes expressed in ESCs and enriched for RNAPII at both the TSS and the gene body were identified by comparison of these datasets. This analysis revealed 3915 genes in GSM1534642, 6318 genes in GSM1566094, and 5133 genes in GSM699166 which were expressed in ESCs and also enriched for RNAPII at the TSS and gene body. These genes were then ranked in order from highest to lowest CHD7 occupancy levels throughout the gene body based on ESC *Chd7* ChIP-Seq dataset GSM558674 (Fig. 4c). The top 500 and bottom 500 CHD7-bound genes were then selected for further calculation of pausing indices (i.e. ratio of RNAPII RPKMs at the TSS (− 50 bp to + 200 bp) to RPKM at the gene body (+ 500 bp to transcription termination site)) (Fig. 4d).

Meta-analysis of all three datasets showed a low pausing index for genes with high CHD7 occupancy in the gene body and a high pausing index for genes with low CHD7 occupancy in the gene body (Fig. 4e). For all three RNAPII ChIP-seq datasets, gene body RPKM for RNAPII were higher for those genes bound by CHD7 than for genes not bound by CHD7 (Fig. 4f). These results suggest a correlation between high CHD7 occupancy and slowed RNAPII processivity in gene bodies. Thus, CHD7 may participate in general chromatin unwinding and remodeling in ESC-derived cells and/or control one or more aspects of transcriptional elongation^18^.

### CHD7 regulates genes expression

CHD7 enrichment in the gene body of ESCs suggests it may alter rates of RNA transcriptional elongation in addition to nascent RNA transcription. Because prior studies of gene expression in *Chd7* mutant cells and tissues focused on steady state RNA, which does not distinguish newly transcribed from older RNA molecules, we asked whether CHD7 may function in ESC-derived NPCs to regulate transcriptional activation, elongation, or both. Bru-Seq is a recently developed large scale sequencing method that uses Bromouridine (Bru) to label newly transcribed RNA^36^. Thus, we used Bru-Seq in *Chd7*^+*/*+^ and *Chd7*^*Gt/Gt*^ NPCs (N = 3) to test the effects of chronic *Chd7* loss on nascent transcription. Principal components plots showing sample clustering, along with summaries of replicates, are presented in Supplementary Fig. 3 and Table 2.

**Figure 5:**
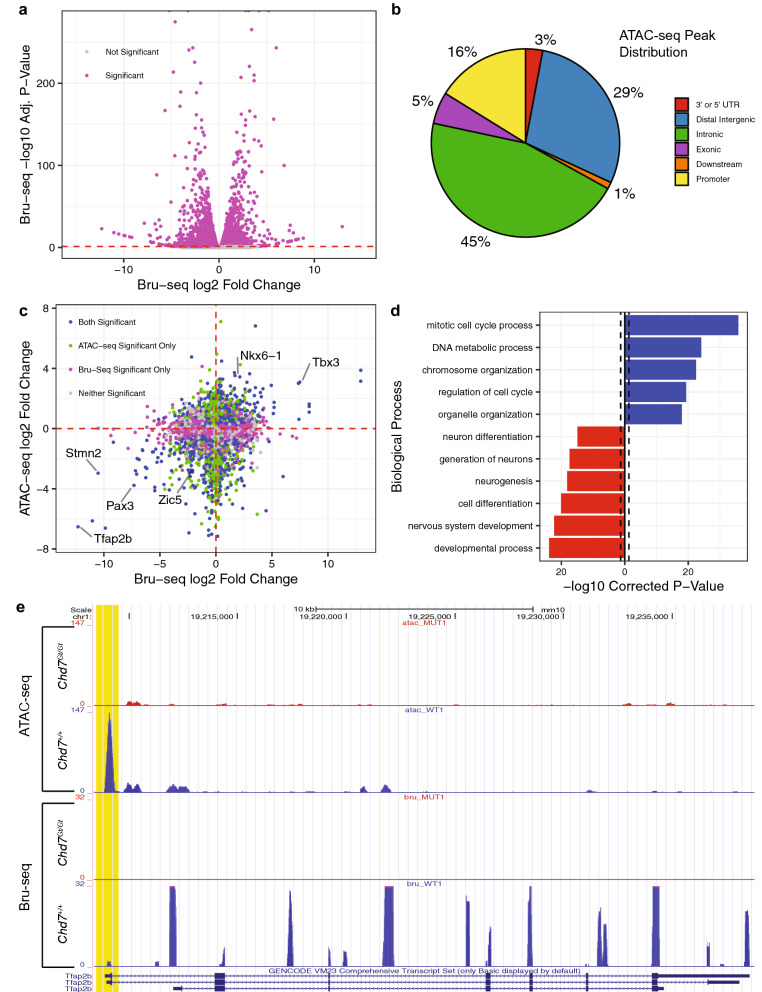
CHD7 loss affects accessibility and expression of key NPC genes. (**a**) Volcano plot showing log2 fold changes and FDRs for genes in the Bru-Seq analysis. (**b**) Pie chart showing the distribution of peak locations from ATAC-seq peaks that were associated with genes expressed in NPCs by Bru-Seq. (**c**) Scatter plot showing peaks from the combined analysis of ATAC-seq and Bru-Seq data. The y-axis is the log2 fold change in peaks. The x-axis is the log2 fold change in gene expression. Peaks are labeled with different colors based on significance thresholds of FDR < 0.05. The upper-right quadrant shows genes with higher accessibility in their promoter region and higher expression in *Chd7*^*Gt/Gt*^ compared to *Chd7*^+*/*+^. The lower left quadrant shows genes with lower accessibility in their promoter region and lower expression in *Chd7*^*Gt/Gt*^ compared to *Chd7*^+*/*+^. (**d**) GO Term analysis of “doubly significant” genes. The p-values were corrected with a Bonferroni adjustment. The black-dashed line shows the cutoff of a corrected p-value of 0.05. Blue bars represent GO terms associated with genes that were up-regulated in *Chd7*^*Gt/Gt*^; red bars represent GO terms associated with down-regulated genes. (**e**) Plot of ATAC-seq and Bru-Seq read tracks along the gene body of *Tfap2b* using the UCSC Genome Browser. The promoter region is highlighted in yellow. The image shows greater accessibility of the promoter region and higher expression in the *Chd7*^*Gt/Gt*^ cell line.

Bru-Seq confirmed higher nascent *Chd7* expression in *Chd7*^+*/*+^ NPCs compared to *Chd7*^*Gt/Gt*^ NPCs, consistent with the overall reduction of *Chd7* observed in *Chd7*^*Gt/Gt*^ ESCs (Fig. 2d). PCA of the Bru-Seq data show a clear separation of genotypes along principal component one and principal component two, indicating consistency across replicates (Supplementary Fig. 5). The total number of genes analyzed in *Chd7*^+*/*+^ and *Chd7*^*Gt/Gt*^ NPCs was 17,346. There were 6,122 differentially expressed genes between *Chd7*^+*/*+^ and *Chd7*^*Gt/Gt*^ NPCs (35%) (FDR < 0.05), with 2,716 (44%) being down-regulated in *Chd7*^*Gt/Gt*^ and 3,406 (56%) being up-regulated in *Chd7*^*Gt/Gt*^ NPCs (Fig. 5a). This result is consistent with previous studies showing that CHD7 both positively and negatively regulates expression of other genes^3,18,27^.

### CHD7 loss disrupts chromatin accessibility at differentially expressed genes

CHD7 uses the energy of ATP hydrolysis to evict, slide, or rearrange nucleosomes on chromatin^16^ . In previous studies, CHD7 binding sites were found in transcriptional start sites as well as intergenic sites and intragenic regions^20^. Most CHD7 binding sites in mouse NPCs have been confirmed as CHD7 binding sites in human cells^20^. To test whether CHD7 modifies the chromatin landscape in mouse NPCs, we used ATAC-seq, an assay for genome-wide mapping of chromatin accessibility, on both *Chd7*^+*/*+^ and *Chd7*^*Gt/Gt*^ NPCs (N = 3) and compared peaks across genotypes. Each peak was associated with a nearby gene and annotated by its location relative to that gene. There were 121,161 combined peaks identified in *Chd7*^+*/*+^ and *Chd7*^*Gt/Gt*^ NPCs. Of these, 99,770 peaks were associated with genes expressed by Bru-Seq assay. Of these peaks, 45% (45,228) were located in intronic regions, 29% (28,876) were in distal intergenic regions, and 16% (16,130) were in promoter regions (Fig. 5b).

To identify misregulated genes whose promoters were also differentially accessible in *Chd7*^*Gt/Gt*^ cells, we further analyzed 12,299 genes that were expressed by Bru-Seq analysis and were associated with at least one ATAC-seq peak in a promoter region. Among these genes, some contained multiple promoter peaks, typically reflecting different transcription start sites for isoforms. Altogether, we observed a total of 16,130 ATAC-seq peaks associated with these 12,299 expressed genes. A scatterplot was created using fold changes for all peak/gene combinations from this combined analysis (Fig. 5c). A Kendall’s tau correlation was performed to determine the relationship between fold change in ATAC-seq peak accessibility and Bru-Seq gene expression between *Chd7*^+*/*+^ and *Chd7*^*Gt/Gt*^ NPCs. There was a significant, positive correlation between ATAC-seq and Bru-Seq fold changes (Kendall’s tau = 0.11, P < 2.2 × 10^–16^), consistent with increased accessibility at the promoter and higher levels of gene expression. Of particular interest were 2,742 differentially expressed genes which were also associated with 3,119 differentially accessible peaks. We call these genes “doubly significant”. GO term analysis of these doubly significant genes was performed for both up- and down-regulated genes. The down-regulated genes (N = 1094, 40%) showed enrichment in many different processes including nervous system development, neurogenesis and cell differentiation. The up-regulated genes (N = 1,648, 60%) showed an enrichment in mitotic cell cycle process, DNA repair and mitotic nuclear division (Fig. 5d).

Review of the top doubly significant genes and significant GO terms (i.e. neurogenesis) revealed several genes of particular interest (Table 1), five of which encode transcription factors (*Tfap2b*, *Pax3, Zic5, Tbx3* and *Nkx6-1*) that control neuronal or glial cell fate^28–34^. Another gene, *Stmn2,* was downregulated in *Chd7*^*Gt/Gt*^ cells and encodes a stathmin protein family member that promotes neuronal growth^35^. These six genes were further analyzed by examination of chromatin accessibility and gene expression using the UCSC Genome Browser. Representative read tracks for *Tfap2b* highlight differences in chromatin accessibility and gene expression (Fig. 5e). All read tracks for *Tfap2b* are shown in Supplementary Fig. 6. Several other doubly significant genes also showed differences in expression and accessibility (Supplementary Fig. 7). Based on these combined genome level data, we speculate that CHD7 alters the chromatin landscape in NPCs by modifying chromatin accessibility at genes that regulate neuron-specific and glial-specific processes.

**Table 1:** Top genes with differential expression and accessibility in *Chd7*^*Gt/Gt*^ NPCs.

Gene	Chr	Peak start position	ATAC-seq		Bru-Seq		Concordance
			log2 FC	Adj. P-value	log2 FC	Adj. P-value	
Stab2	10	86,471,533	3.87	1.34E−03	12.93	3.45E−26	Yes
Stab2	10	86,470,475	3.15	1.06E−05	12.93	3.45E−26	Yes
Pax2	19	44,830,636	1.09	6.01E−06	8.32	5.73E−11	Yes
Pax2	19	44,829,214	1.63	6.87E−03	8.32	5.73E−11	Yes
Pax2	19	44,830,054	1.47	4.19E−03	8.32	5.73E−11	Yes
Tbx3	5	120,120,208	3.08	3.81E−121	7.48	6.68E−28	Yes
Lhx4	1	157,589,145	0.62	1.23E−02	7.41	3.07E−08	Yes
Zdbf2	1	63,319,600	3.01	2.40E−02	7.36	1.80E−21	Yes
Pax8	2	24,301,469	− 3.19	1.57E−02	6.00	6.91E−244	No
Grin2b	6	136,123,298	1.41	1.11E−06	5.20	0.00E+00	Yes
Nkx6-1	5	102,093,567	3.52	2.85E−178	1.70	8.59E−29	Yes
Zic5	14	122,863,053	− 2.93	4.23E−100	− 2.38	1.67E−06	Yes
Map10	8	128,193,658	− 2.06	3.04E−02	− 6.86	4.80E−07	Yes
Eef1a2	2	180,892,656	− 1.99	1.69E−02	− 7.03	1.00E−06	Yes
Clec12b	6	129,334,486	− 3.03	1.20E−39	− 7.08	6.61E−07	Yes
Hoxc4	15	102,864,898	− 2.80	7.89E−29	− 7.18	7.74E−08	Yes
Pax3	1	78,192,985	− 3.77	2.97E−66	− 7.31	5.81E-16	Yes
Pitx2	3	128,902,161	− 0.91	9.44E−03	− 9.15	3.97E−13	Yes
Mab21l1	3	55,586,203	− 6.61	4.50E−16	− 9.87	4.29E−15	Yes
Stmn2	3	8,509,214	− 2.96	1.54E−08	− 10.52	3.57E−17	Yes
Phox2b	5	67,489,831	− 6.14	2.11E−101	− 11.03	7.30E−19	Yes
Tfap2b	1	19,198,995	− 6.53	9.69E−26	− 12.31	1.27E−23	Yes

## Discussion

This study demonstrates that CHD7 is dispensable for maintenance/survival of mouse ESC pluripotency and differentiation to neural progenitor cells yet promotes differentiation of neurons and glia from embryonic stem cell-derived NPCs. CHD7 also promotes neurite length, branching, and complexity in ESC-derived neurons, and regulates gene expression and chromatin accessibility at key neural and glial developmental genes. This developmental stage-specific requirement for CHD7 may reflect the need for a changing chromatin landscape as cells transition from pluripotent to lineage restricted cells, highlighting unique functions for CHD7 in later stages of the neuronal/glial differentiation process.

We found no evidence for defects in growth (cell number) in *Chd7*^*Gt/Gt*^ ESCs or NPCs, suggesting that CHD7 is not necessary for ESC or NPC proliferation. Interestingly, individuals with CHARGE syndrome and mouse models of CHD7 deficiency show cerebellar hypoplasia but not microcephaly or macrocephaly, and CHD7 promotes granule cell, but not cortical neural progenitor cell, proliferation^3,18,36^. In the mouse cerebellum, loss of *Chd7* leads to decreases in the number of dividing granule cell precursors^3,18^. In contrast, conditional knockout of *Chd7* in hippocampal glia and neural progenitors is associated with a transient increase in cell division and genesis of immature neurons, with depletion of the stem cell pool in the sub-granular zone^12^. Germline and conditional *Chd7* knockout mice also show decreased neurogenesis in the adult sub-ventricular zone and hippocampus^10,11^, dentate gyrus^12^, and inner ear^15^. In non- neuronal cell types like ESCs, however, loss of *Chd7* has no impact on cell growth^19^. These observations together support the conclusion that CHD7 can promote, inhibit, or have no effects on cell proliferation, depending on the context and cell type.

Apoptosis is key to proper organismal development and tissue maintenance^37^ and involves similar cellular processes as differentiation^10^. We observed very few apoptotic ESCs or NPCs in either *Chd7*^+*/*+^ or *Chd7*^Gt/Gt^ cells, and no difference in the number of apoptotic cells between genotypes. Contrary to this finding, loss of CHD7 is associated with increased cell death in mouse cerebella as measured by the number of Caspase-positive cells^3,18^. Differences in apoptosis rates across studies may reflect differing sensitivities in neuronal cell type to *Chd7* loss.

We observed changes in expression of ESC-related genes like *Oct4*, *Sox2,* and *Nanog* between *Chd7* null and wild-type ESCs. It is unclear why these pluripotency markers differ in expression between *Chd7*^+*/*+^ and *Chd7*^*Gt/Gt*^ cells, but this could reflect direct regulation of CHD7 on the promoters of these genes, or other subtle forms of regulation. Schnetz et al*.* reported normal mRNA levels for *Oct4*, *Sox2*, and *Nanog* in *Chd7* null ESCs, and showed that loss of *Chd7* does not preclude ESC pluripotency and differentiation into embryoid bodies^19^. Differences between their study and ours may be related to the underlying genetic defect (nonsense vs gene-trapped alleles), genetic background, or other experimental conditions. Interestingly, CHD7 co-localizes with OCT4, SOX2, NANOG and P300 in mouse ESCs, and *Oct4* is required for CHD7 binding to genomic segments that are bound by multiple transcription factors^13,19,38^. Moreover, loss of *Chd7* does not impair advancement of embryonic development to the blastocyst stage from which ESCs are generated, since null embryos die after E10.5^38^. Together, these observations support the conclusion that *Chd7* is dispensable for ESC pluripotency and self-renewal.

In contrast to these early stages of development where *Chd7* is not absolutely required, later stages of lineage formation and gene expression have been shown to depend on CHD7. For example, downregulation of CHD7 disrupts EB formation and expression of endoderm and mesoderm genes^39^. In our study, *Chd7*^Gt/Gt^ ESCs were able to differentiate normally into PAX6-positive NPCs, but *Chd7*^Gt/Gt^ NPCs showed reduced ability to differentiate into TUJ1-positive neurons. Similarly, in previous studies, neural stem and progenitor cells from the sub-ventricular zone of *Chd7*^+*/−*^ and *Chd7*^*−/−*^ mice showed reduced potential to differentiate into TUJ1-positive neurons^11^. Additionally, loss of *Chd7* in mice leads to fewer neurons in the olfactory bulb^11^. Thus, normal levels of CHD7 are required to maintain multiple neuronal lineages in a variety of peripheral and central nervous system sites^22^. Our results showed that the number of ESC-derived neurons expressing NEUN, MAP2, Calretinin or Neurofilament-200 was unchanged with *Chd7* loss, suggesting that only certain aspects of neuronal differentiation require *Chd7*. We cannot exclude the possibility that certain neuronal subtypes (e.g. TUJ1 +) or developmental stages (e.g. early differentiation) are more sensitive to *Chd7* loss than others. Indeed, we observed defective neurite branching number, length, and complexity in *Chd7*^*Gt/Gt*^ neurons in vitro, both at 5 and 9 days in culture (Fig. 4), and similar defects in neurite length and branching were reported in newly generated hippocampal neurons with loss of *Chd7*^10^, consistent with our findings. It will be interesting to determine in future studies whether specific neuronal subtypes are preferentially affected by *Chd7* loss. Our analysis of existing RNAPII and CHD7 ChIP-Seq data in ESCs showed enrichment of RNAPII in genes that are also occupied by CHD7. This analysis also uncovered lower RNAPII pausing indices for genes with high CHD7 occupancy. While correlative, these data suggest a model whereby CHD7 promotes release of RNAPII from the transcription start site and regulates the rate at which RNAPII travels through the gene body during transcription. Additional studies using controlled, inducible, CHD7 and RNAPII expression will help explore this possibility. It will also be helpful to determine whether enrichment of CHD7 in the gene body of highly expressed genes also occurs in differentiated cells like neurons and glia.

Our combined analysis using Bru-Seq and ATAC-seq on *Chd7*^+*/*+^ and *Chd7*^*Gt/Gt*^ NPCs identified many genes whose expression and accessibility were significantly altered by *Chd7* loss. Several of these genes are implicated in neural progenitor cell differentiation, and the biological processes (based on GO terms) associated with these gene expression changes include nervous system development and differentiation. One gene, *Pax3*, exhibited a striking loss of open chromatin near the promoter, and reduced expression in *Chd7*^*Gt/Gt*^ NPCs. *Pax3* has been shown to directly bind and regulate *Hes1* and *Neurog2*, thereby promoting differentiation of sensory neurons^40^. Uncovering the extent to which *Pax3* and other CHD7-sensitive genes mediate the effects of *Chd7* in neural progenitors will require new methods that allow for simultaneous up- and down-regulation of multiple genes and genetic pathways. Such approaches will help clarify not only which genes and pathways are critical for CHD7 function but will also enable the identification of putative therapeutic targets.

Our studies also identified *Stmn2*, the gene encoding phosphoprotein stathmin family member 2, as a putative target of CHD7. STMN2 regulates microtubules and neuronal growth, is highly expressed in the developing brain, and is downregulated in postmortem brains from individuals with Parkinson’s Disease^41^. *Stmn2* has been shown to function in synaptic trafficking and is necessary for axonal outgrowth and regeneration^41^, akin to the changes in complexity we observed in neurons lacking *Chd7*. Reduced *Stmn2* expression may help explain the defects in neuronal complexity and neurite outgrowth observed with loss of *Chd7* in ESC-derived neural and hippocampal cells.

We also observed reduced expression of *Tfap2b*, encoding transcription factor AP2-β in *Chd7*^*Gt/Gt*^ ESC-derived neural cells. Pathogenic variants in *TFAP2B* cause human CHAR syndrome, an autosomal dominant disorder that presents in individuals with patent ductus arteriosus, facial dysmorphisms, and abnormalities of the 5th digits of the hands^42^. Some features of CHAR overlap with CHARGE, include developmental delay, broad forehead, short philtrum, low-set ears, and congenital heart disease. Of note, TFAP2B has been shown to promote differentiation of neural crest cells via heterodimerization with the pioneer transcription factor and family member TFAP2A^43^. Since neural crest cells contribute to craniofacial, cardiac, and skeletal structures, this cell population is implicated in both CHAR and CHARGE syndromes and could be considered as a candidate target population for therapeutics^44,45^.

In conclusion, this study provides the first evidence for CHD7 regulation of nascent transcription and chromatin accessibility in ESC-derived neural progenitors. We present several candidate genes as putative genetics targets for CHD7 that merit further analysis, including *Tfap2b, Stmn2, Sox1, Pax3, Zic5, Tbx3,* and *Nkx6-1*. These genes may mediate CHD7 effects in neuronal and glial differentiation, and a genome-wide, combinatorial approach will be helpful to further address this possibility. Our results suggest that therapies directed toward correction of human phenotypes of *CHD7* deficiency, particularly those affecting the central and peripheral nervous systems, may also need to target multiple genetic pathways during early stages of cellular differentiation, rather than focusing on stem or progenitor cell stages.

## Materials and methods

All reagents and culture media were purchased from Life Technologies/Invitrogen (Carlsbad) unless otherwise indicated. Methods were carried out in accordance with relevant guidelines and regulations, and experimental protocols involving mice (generation of embryonic stem cells from blastocysts) were approved by the University of Michigan Institutional Animal Care & Use Committee (IACUC).

### Western blotting

Nuclear protein was extracted and quantified using the BCA Protein Assay Kit (Life Technologies). Protein samples (10–20 µg) were electrophoresed in 4–12% Bis–Tris gels and transferred overnight to a nitrocellulose membrane at 4 °C. Primary antibodies used were anti-HDAC2 (Santa Cruz) and anti-CHD7 (#6505, Cell Signaling). Secondary antibodies were goat anti-rabbit IgG HRP (#31461, Thermo Fisher) and rabbit anti-goat IgG HRP (#31403, Thermo Fisher).

### Neuronal lineage differentiation

*Chd7*^+*/*+^ and *Chd7*^*Gt/Gt*^ ESCs were derived from sibling mouse blastocysts in the Transgenic Animal Model Core (TAMC) of The University of Michigan. *Chd7*^+*/*+^ (passage 10) and *Chd7*^*Gt/Gt*^ (passage 10) cells were sent to Cell Line Genetics (Madison, Wisconsin, US) for karyotyping based on cytogenetic analysis on G-banded metaphase cells. Both cell lines were > 85% euploid and were used only up to passage 20–21.

ESCs were cultured in knockout DMEM, 20% FBS (Fetal bovine serum, Atlas Biologicals, Fort Collins, CO), Glutamax, MEM Nonessential amino acids, 2-Mercaptoethanol, and Recombinant Mouse Leukemia Inhibitory Factor (LIF) without feeder cells. ESCs were differentiated to neuronal cells following an established protocol^46^. Mouse ESCs were dissociated to single cells using 0.05% Trypsin–EDTA, then seeded at a density of 3 × 10^5^ cells /ml in embryoid body (EB) medium (withdrawal of LIF) onto bacteriological dishes. Cells were cultured as aggregates in suspension. EBs formed after 4 days, then 5 μM ATRA was added to the medium and cultures allowed to grow for 4 more days. EBs were dissociated and plated onto poly-L-Lysine (Sigma) coated coverslips in 6-well-plates at 1 × 10^5^ cells per well in NPC medium (10 ng/ml basic fibroblast growth factor, 1% N2, 1% Penicillin/Streptomycin, supplemented by DMEM/F12). Neurospheres were maintained in DMEM/F12 supplemented with B27, 20 ng/ml EGF, 20 ng/ml bFGF, Glutamax and Penicillin/ Streptomycin for cell line establishment. NPCs were harvested for immunofluorescence for PAX6, NESTIN, VIMENTIN and SOX2.

Cells were cultured in NPC medium for 3 days, then switched to neuronal media (1 mM Glutamax, 1% Penicillin/ Streptomycin, 1% N2, 2% B27, supplemented with DMEM/F12) and differentiated into neurons and glia^46^. Cells were incubated at 37 °C with 5% CO_2_ and cultured on plates coated with poly-L-lysine. The medium was changed every other day. Neural cells were harvested on day in vitro (DIV) 5 for immunostaining with anti-TUJ1, GFAP, NEUN, MAP2, Calretinin and Neurofilament-200 (NF200).

### Quantitative RT-PCR

Cells (at least 10^6^) were collected by centrifugation, lysed by cold Trizol and incubated for 5 min to ensure complete lysis. An equal volume of Chloroform (Thermo Fisher Scientific) was added to the lysis product and vortexed for 15 s. After centrifugation, the supernatant was removed and added to the same volume of Isopropanol (Thermo Fisher Scientific) to precipitate RNA. After centrifugation, the supernatant was discarded, and the pellet washed with 70% DNase/RNase free ethanol solution. After drying for 5 min, RNA was dissolved in 20 µl nuclease free H_2_O.

cDNA was synthesized using the SuperScript First-Strand Synthesis System kit (Thermo Fisher Scientific) and stored at − 20 °C. DNA samples were quantified using a Nanodrop 2000c (Thermo Fisher Scientific). qRT-PCR experiments were performed using SYBR Green Master Mix (Applied Biosystems, Foster City, CA) on an ABI 7500 qPCR machine (Applied Biosystems).

Primers are shown in Supplementary Table 1. Gene expression was normalized to *Gapdh*. Statistical significance was determined using unpaired, two-tailed Student’s *t*-tests. P-values were corrected using the Holm-Sidak method^47^.

### Cell growth assays

For cell growth assays, 50,000 ESCs or 100,000 NPCs were plated per well in a 6-well plate. Wells were coated with 0.1% gelatin for ESCs or with poly-L-lysine (Invitrogen, Carlsbad, CA) for NPCs. Cells were maintained in culture media for the length of the assay. Cell numbers were assayed every two days. Colonies were digested to single cells with 0.05% trypsin–EDTA for ESCs and Accutase for NPCs. Cells were diluted in culture media and counted with a hemocytometer. Statistical significance was determined using unpaired, two-tailed Student’s t-tests. P-values were corrected using the Holm-Sidak method^47^.

### Immunostaining

Cells were plated on poly-l-lysine coated coverslips, washed three times with PBS, then fixed in 4% paraformaldehyde (PFA, Sigma) in phosphate buffer (pH 7.4) for 30 min at room temperature. Cells were stored at 4 °C in 4% paraformaldehyde or immediately used for immunostaining. Coverslips were washed with PBS three times, then treated with 0.1% Triton-X-100 (Sigma) for 15 min. Coverslips were blocked with 2% bovine serum albumin (BSA) for one hour and incubated with primary antibodies: rabbit anti-PAX6 antibody (1:300, #901301, Biolegend, San Diego, CA), rat anti-Nestin antibody (1: 200, #rat-401, Developmental Studies Hybridoma Bank, Iowa City, IA), goat anti-Vimentin, (1:100, #V4630, Sigma-Aldrich, St. Louis, MO), goat anti-SOX2 (1:200, #GT15098, Neuromics, Edina, MN), mouse anti-Tuj1 (1:200, #801202, Biolegend, San Diego, CA), mouse anti-NEUN (1:100, #MAB3777, Millipore Billerica, MA), mouse anti-MAP2 (1:200, #M4403, Sigma Aldrich St. Louis, MO), rabbit anti-Neurofilament 200 (1:100, #N4142, Sigma Aldrich St. Louis, MO), rabbit anti-Calretinin (1:100, #AB5054, Sigma Aldrich St. Louis, MO), rabbit anti-cleaved Caspase 3 (1:200, Cell Signaling, Danvers, MA), and mouse anti-GFAP (1:200, #G6171, Sigma Aldrich St. Louis, MO) overnight at 4 °C. After incubation with primary antibody, cells were washed three times with PBS, then incubated with secondary antibody for 2 h at room temperature. Secondary antibodies used were as follows: goat anti-rabbit IgG H&L Alexa Fluor 555 (1:300, #ab150078, Abcam), goat anti-mouse IgG H&L Alexa Fluor 555 (1:500, #ab150118, Abcam), donkey anti-goat Alexa Fluor 488 (1:200, #ab150129, Abcam), and donkey anti-mouse Alexa Fluor 488 (1:200, #ab150105, Abcam). Coverslips were washed with PBS (3 × 10 min) and incubated with 1:10,000 4′,6-diamidino-2-phenylindole (DAPI; Thermo Fisher Scientific, Waltham, MA) for 5 min. Samples were washed with PBS and mounted with Prolong Gold Antifade reagent. Cells were imaged using a Leica DMB upright fluorescence microscope. Statistical significance was determined using paired, two-tailed Student’s *t*-tests.

### Sholl analysis

Neural cells were harvest at DIV5 and DIV9 and immunostained with anti-MAP2 as described above. The longest neurite for each MAP2 positive cell was measured using Sholl analysis, as previously described^48^ with Fiji software (National Institutes of Health, Bethesda, MD, USA). Sholl analysis of single neuron branching was performed manually using Simple Neurite Tracer.

### Bru-Seq

*Chd7*^+*/*+^ and *Chd7*^Gt/Gt^ mouse ESC and NPC cells were collected and at least 6 × 10^6^ cells for genotype were used for the Bru-Seq analysis as previously reported^42^. Briefly, cells in the logarithmic growth phase were treated with bromouridine to a final concentration of 2 mM for 30 min to label newly synthesized nascent RNA. Cells were then lysed in TRIzol (Invitrogen) and total RNA was isolated. Bru-labeled, nascent RNA was then immunocaptured using anti-BrdU antibodies (BD Biosciences, San Jose, CA, USA) conjugated to magnetic beads (Invitrogen) and then converted into cDNA libraries and sequenced at the University of Michigan Sequencing Core using an Illumina HiSeq 2000 sequencer (San Diego, CA). Sequencing reads were mapped to the mm10 reference sequence and the relative rates of nascent transcription genome-wide were calculated as previously described^42^.

### ATAC-seq

*Chd7*^+*/*+^ and *Chd7*^*Gt/Gt*^ neural progenitor cells (differentiated from ESCs) were collected according to protocol^49^. Briefly, 50,000 cells for each genotype were collected and lysed in 50 µl cold ATAC-Resuspension Buffer containing 0.1% NP40 (Sigma), 0.1% Tween-20 (Sigma), and 0.01% Digitonin (Promega). Lysis product was incubated with Tn5 transposase (Illumina, Nextera XT DNA Library Prep Kit) for 30 min at 37 °C. cDNA from Tn5-treated cells was purified and used as a template to amplify a DNA library by PCR with 2 × NEB master mix. Libraries were deep-sequenced with Next-seq 500 and high output kit (75 bp) with paired-end 36 bp reads. Three replicates for each cell line were collected and processed.

ATAC libraries were prepared as described previously^50^. In brief, 50,000 cells per sample were processed for the transposase reaction. ATAC-seq libraries were PCR amplified with barcoded primers. ATAC-seq libraries were sequenced (paired-end 36 bp on a HiSeq4000at the UM Sequencing Core) and raw reads trimmed using cta (https://github.com/ParkerLab/cta)^50^ to eliminate barcodes and aligned to the mm9 reference genome using BWA-MEM (v. 0.7.15)^51^. Picard was used to remove duplicates, and reads were subsequently filtered for high mapping quality (mapq ≥ 30) and properly paired alignments, as described^52^. In order to minimize potential confounding by sequencing depth, libraries were uniformly down sampled to the minimum sequencing depth (31.9 M reads) for downstream analysis steps.

Peaks were called using MACS2^53^ with options *–nomodel –shift -100 –extsize 200 –broad -B –keep-dup all* on the down sampled BAM files. Peak calls that met a 5% False Discovery Rate and did not intersect genomic regions with known mappability artifacts (blacklisted regions from https://sites.google.com/site/anshulkundaje/projects/blacklists) were retained for further analyses. A virtual session of the quality control metrics for the ATAC-seq samples is provided at the following URL: https://theparkerlab.med.umich.edu/data/albanus/martin/ataqv_npc/.

There were 88,641 peaks in the *Chd7*^+*/*+^ NPCs and 81,773 peaks in the *Chd7*^*Gt/*Gt^ NPCs. The peak regions from *Chd7*^+*/*+^ and *Chd7*^*Gt/Gt*^ cells were merged together to obtain a common set of peaks for NPCs, in order to compare peak reads across genotypes. The coverage from each replicate was then determined for the combined peaks. This analysis was done on Python (v. 2.7.14 Anaconda) with Bedtools (v. 2.25.0)^54^. The raw counts were then merged into a matrix which was analyzed in R (v. 3.5) using DESeq2 (v. 1.20.0)^55^. Peaks were matched to genes with ChIPseeker (v. 1.18.0)^56^ with a promoter definition of 1000 bp upstream to 1 bp downstream from the transcription start site^57^.

### UCSC genome browser

Sequencing tracks were generated for the ATAC-seq and the Bru-Seq in bigwig format. These files were then uploaded and viewed in the UCSC Genome Browser, using the mm10 assembly^58^.

### GO term analysis

The Generic Gene Ontology (GO) Term Finder developed at the Lewis-Sigler Institute at Princeton University^59^ was used to analyze the data. All enrichment calculations were done using only expressed genes from the Bru-Seq analysis as the background.

### Statistical analysis

Data were analyzed using GraphPad Prism and R. Unless otherwise indicated, graphs show mean and standard deviations. Unpaired *t-*tests were used and were adjusted for multiple comparisons with the Holm-Sidak method, unless otherwise indicated. P-value adjustment with the Benjamini–Hochberg method was used to calculate the upper bound for the expected false discovery rates (FDR). Kendall’s tau coefficient was used to measure the ordinal association between measured quantities due to a lack of normality in the data. All statistical tests were two-sided.

## Supplementary information

Supplementary Information.

## Data Availability

The datasets generated and/or analyzed during the current study are available from the corresponding author upon request.
