# Supplementary material for: CHD7 promotes neural progenitor differentiation in embryonic stem cells via altered chromatin accessibility and nascent gene expression
Source: Sci Rep. 2020 Oct 15;10:17445. doi: 10.1038/s41598-020-74537-4 (PMC7562747; doi:10.1038/s41598-020-74537-4)
Supplement: Supplementary file 1 — Supplementary Information. [file 41598_2020_74537_MOESM1_ESM.pdf]

**Supplementary Information**

**Title:** CHD7 promotes neural progenitor differentiation in embryonic stem cells via altered chromatin accessibility and nascent gene expression

Hui Yao[1,§], Douglas F. Hannum[2,§], Yiwen Zhai[1,3], Sophie F. Hill[4], Ricardo D'Oliveira Albanus[5], Wenjia Lou[1], Jennifer M. Skidmore[1], Gilson Sanchez[1], Alina Saiakhova[6], Stephanie L. Bielas[7], Peter Scacheri[6], Mats Ljungman[8], Stephen C. J. Parker[5,7] and *Donna M. Martin[1,7]

§Co-first authors

Department of Pediatrics[1], Department of Biostatistics[2], College of Literature, Science, and the Arts[4], Department of Computational Medicine and Bioinformatics[5], Department of Human Genetics[7], Department of Radiation Oncology[8], University of Michigan, Ann Arbor, MI; Center of Genetic and Prenatal Diagnosis[3], The First Affiliated Hospital of Zhenzhou University; Department of Genetics and Genome Sciences[6], Case Western Reserve University, Cleveland, OH

Running title: CHD7 promotes ESC neuronal differentiation

Keywords: CHD7, neuronal differentiation, ATAC-seq

*Correspondence to:

Donna M. Martin, M.D., Ph.D.

1150 W. Medical Center Dr.

8220C MSRB III

Ann Arbor, MI 48109-5652

donnamm@umich.edu

TEL: (734) 647-4859

FAX: (734) 763-9512

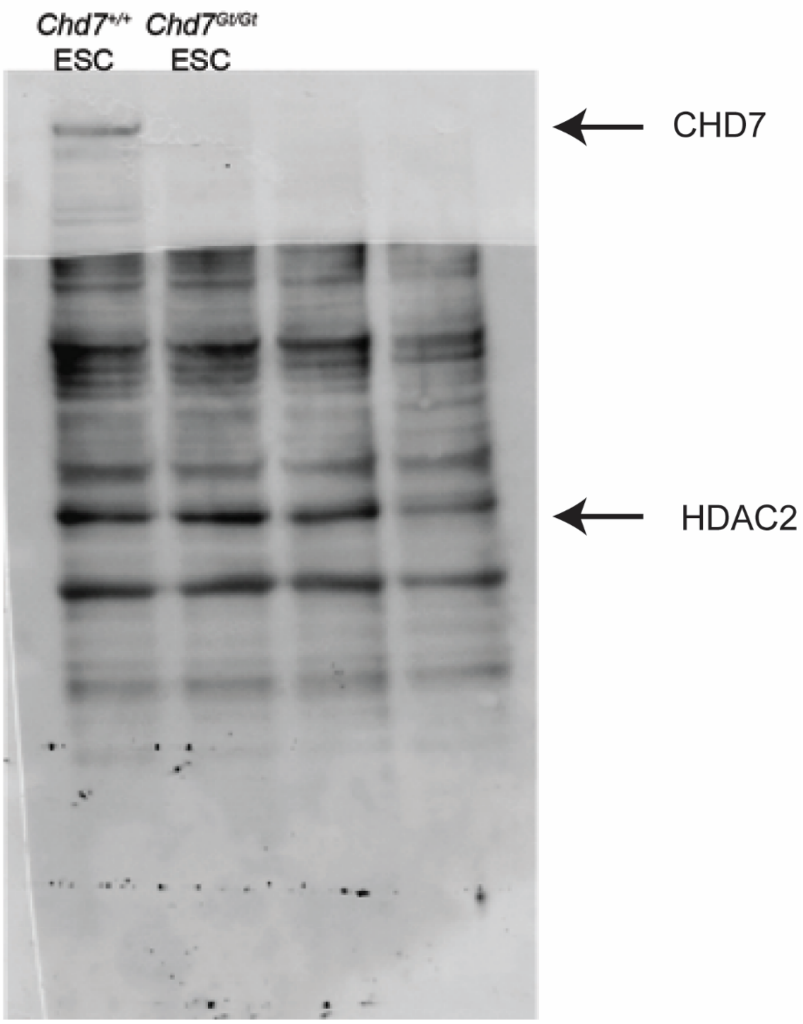


**Supplementary Figure 1. Western Blot for CHD7**

Western blot of the *Chd7*$^{+/+}$ and *Chd7*$^{Gt/Gt}$ ESCs confirming lack of CHD7 in the *Chd7*$^{Gt/Gt}$ cell line, using HDAC2 as a control.

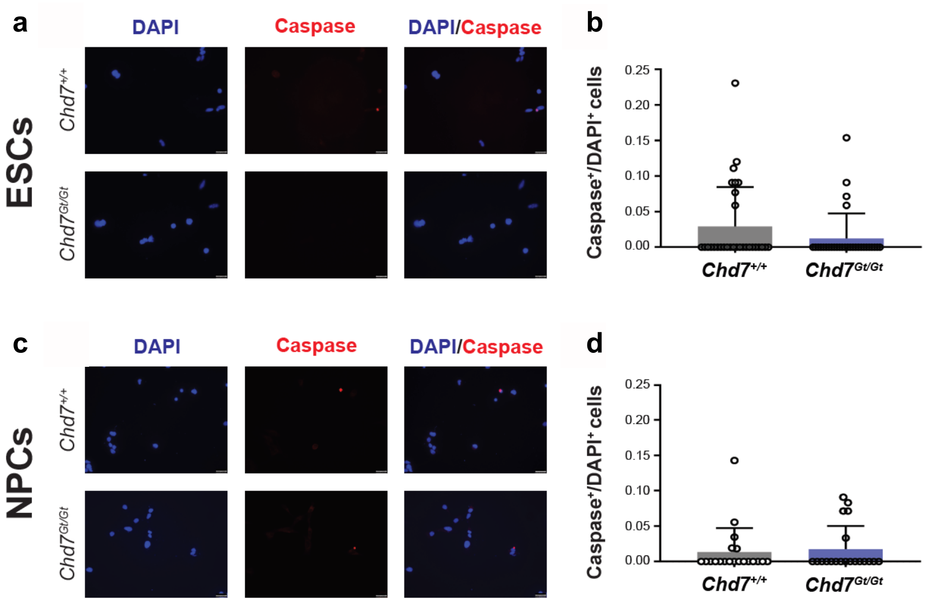


**Supplementary Figure 2. Apoptosis is unchanged with loss of *Chd7***

(a, c) Representative images of cleaved Caspase 3 immunostained *Chd7*$^{+/+}$ and *Chd7*$^{Gt/Gt}$ embryonic stem cells (ESCs) (N = 30) and neural progenitor cells (NPCs) (N = 20), with quantification (b, d) of images.

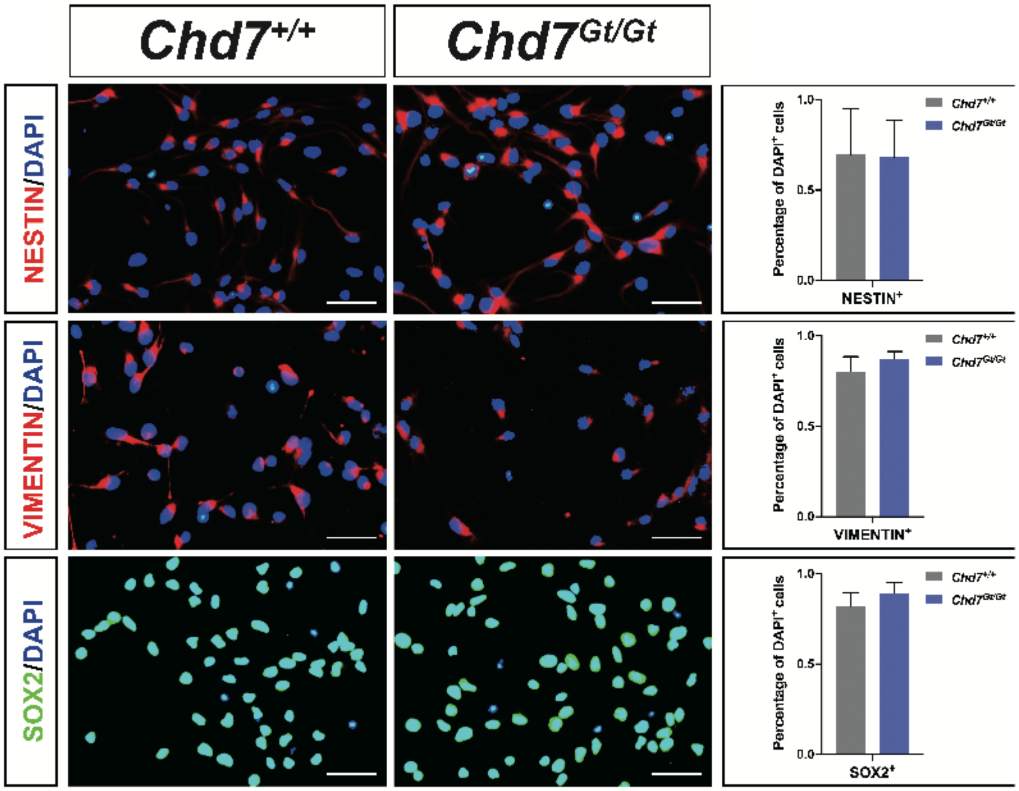


**Supplementary Figure 3. Immunostaining of neuronal progenitor markers.**

Immunostaining for the neuronal progenitor markers NESTIN, VEMENTIN and SOX2 in cells derived from *Chd7*$^{+/+}$ and *Chd7*$^{Gt/Gt}$ ESCs (left). Quantification of immunostaining (right) shows no significant differences between *Chd7*$^{+/+}$ and *Chd7*$^{Gt/Gt}$ in the proportion of NESTIN$^{+}$ cells, VIMENTIN$^{+}$ cells, and SOX2$^{+}$ cells. Data from three independent experiments are represented as mean ± SEM. Unpaired student's *t*-tests were used for statistical analysis. Scale bars = 50 μm.

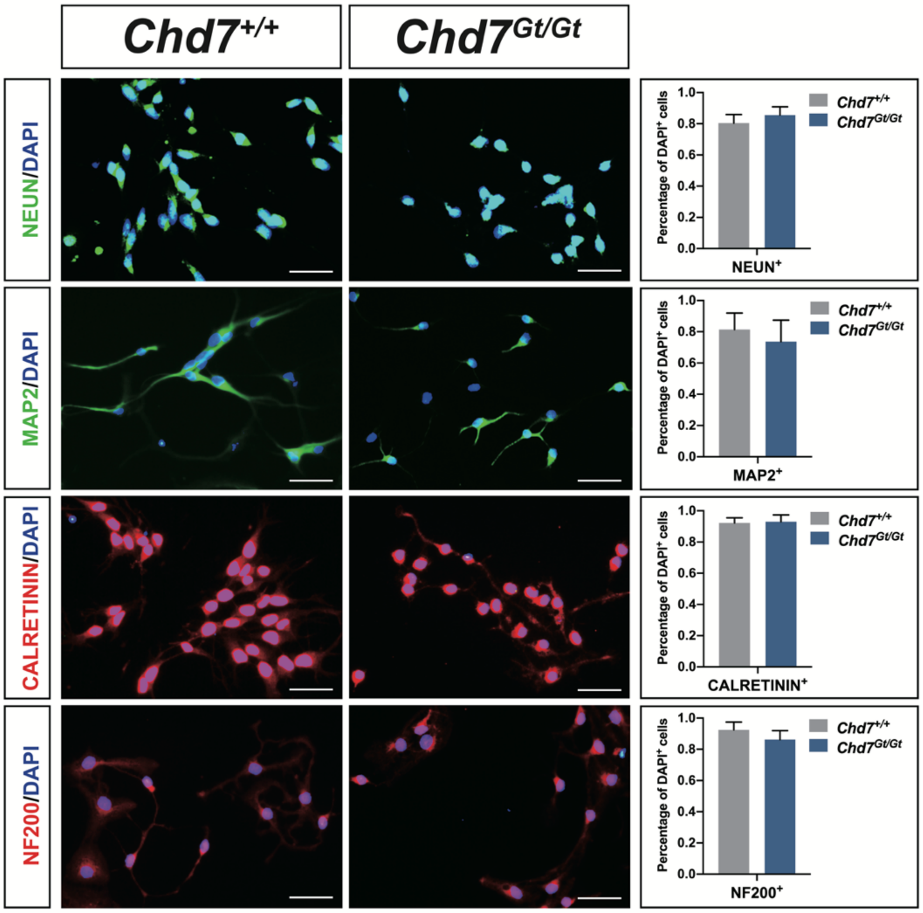


**Supplementary Figure 4. Immunostaining of neuronal markers.**

Immunostaining for the neuronal markers NEUN, MAP2, CALR and NF200 in cells derived from *Chd7*$^{+/+}$ and *Chd7*$^{Gt/Gt}$ NPCs (left). Quantification of immunostaining (right) shows no difference between *Chd7*$^{+/+}$ and *Chd7*$^{Gt/Gt}$ in the proportion of NEUN$^{+}$ cells, MAP2$^{+}$ cells, CALR$^{+}$ cells, and NF200$^{+}$ cells. Data from three independent experiments are represented as mean ±SD. Unpaired student's *t*-tests were used for statistical analysis. Scale bars = 50 μm.

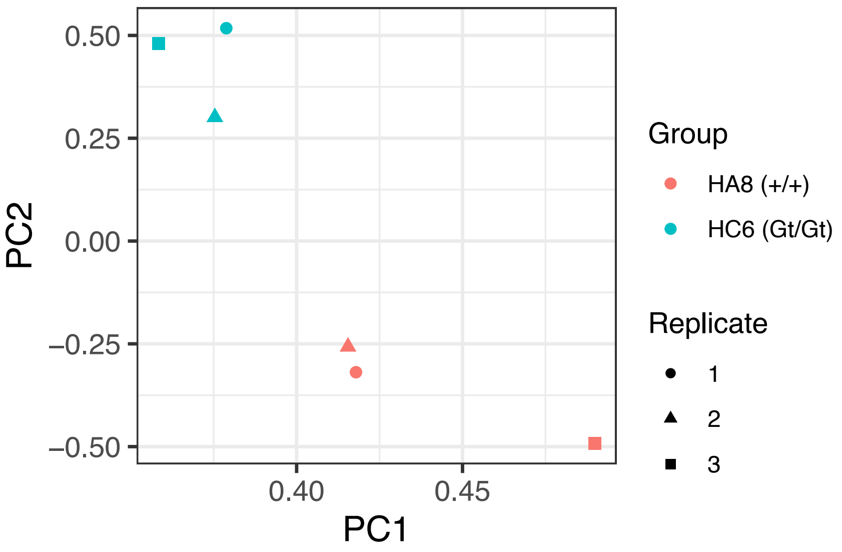


**Supplementary Figure 5. Principle component analysis of Bru-Seq data**

Graphical representation of the first and second principal components for the six Bru-Seq samples. Colors indicate the two different genotypes: HA8 (+/+) is *Chd7*$^{+/+}$ and HC6 (Gt/Gt) is *Chd7*$^{Gt/Gt}$. Shapes represent the different replicates for each genotype. Groups cluster separately from each other.

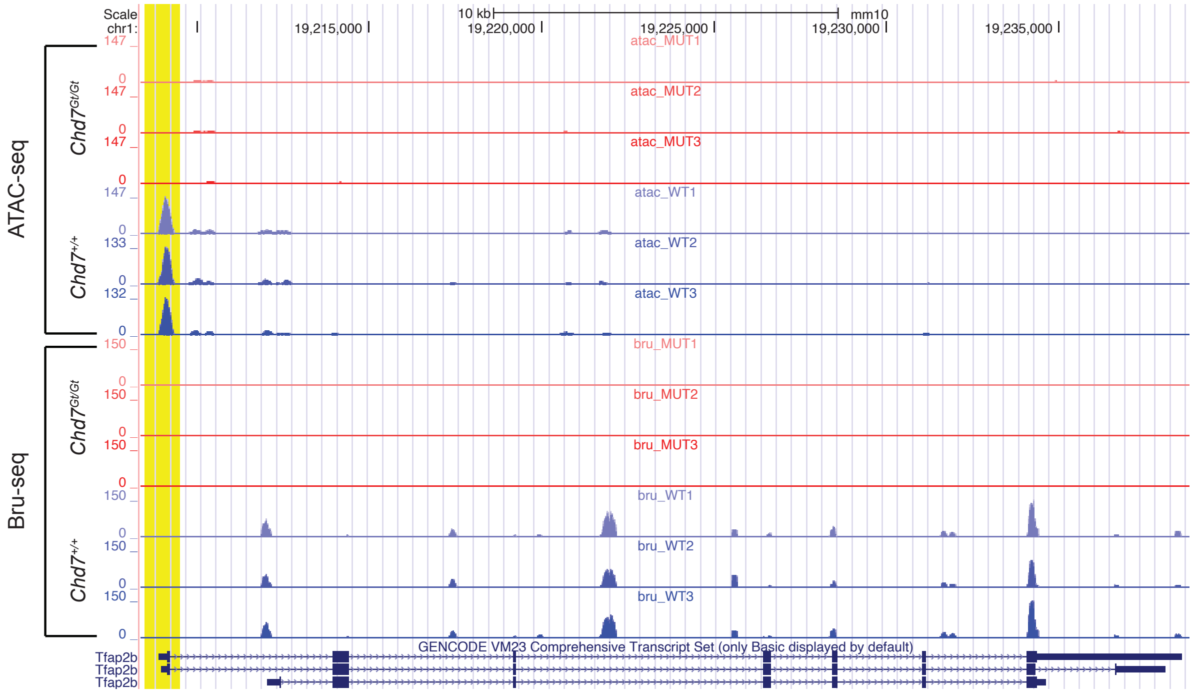


**Supplementary Figure 6. UCSC Genome Browser Tracks for *Tfap2b***

Image showing all tracks for the 3 replicates for Bru-Seq and ATAC-seq data at the *Tfap2b* locus. Yellow highlight indicates the promoter region showing differential accessibility between genotypes.

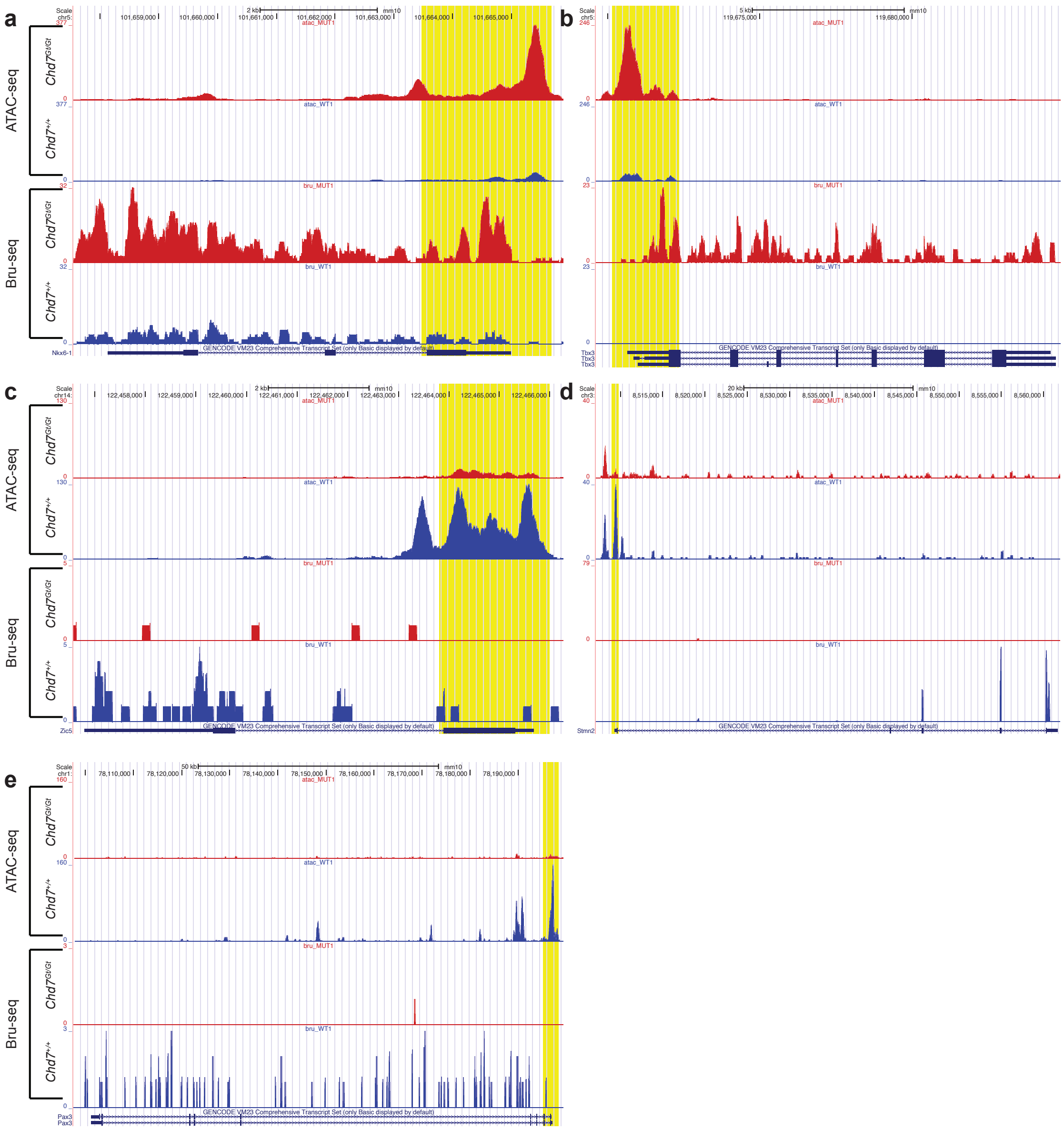


**Supplementary Figure 7. Representative UCSC Genome Browser Images**

(a-e) Images showing reads for the ATAC- and Bru-Seq experiments for (a) *Nkx6*-1, (b) *Tbx3*, (c) *Zic5*, (d) *Stmn2* and (e) *Pax3* as displayed in the UCSC Genome Browser. Promoter regions are highlighted in yellow. The ATAC-seq reads show a visible difference between *Chd7*$^{+/+}$ and *Chd7*$^{Gt/Gt}$, with subtler changes in expression by Bru-Seq analysis.

**Supplementary Table 1. Primers Used for RT-qPCR**

| Gene/locus | Direction | Primer sequence (5' to 3') |
|---|---|---|
| *Oct4* | Forward | TCT TTC CC CAG GCC CCC GGC TC |
| | Reverse | TGC GGG CGG ACA TGG GGA GAT CC |
| *Sox2* | Forward | TTC GAG GAA AGG GTT CTT GCT G |
| | Reverse | TCC TTC CTT GTT TGT AAC GGT CCT |
| *Nanog* | Forward | AGG GTC TGC TAC TGA GAT GCT CTG |
| | Reverse | CAA CCA CTG GTT TTT CTG CCA CCG |
| *C-Myc* | Forward | TGA CCT AAC TCG AGG AGG AGC TGG |
| | Reverse | AAG TTT GAG GCA GTT AAA ATT ATG GCT GAA GC |
| *Brn2* | Forward | AGA GCC CAA GGC AGA AAA GT |
| | Reverse | GGC GCT CTG GTT AAA GGA G |
| *Gpm6a* | Forward | CTT GGA TCT GCG TCA GTT TG |
| | Reverse | GGA AGT TCT CAG AGG CAG TAC AA |
| *Ncan* | Forward | GGA CAG ACA ACA CAG GAC TGC |
| | Reverse | CCC ACC TGC GAA GAA ATT AT |
| *Olig1* | Forward | AGG GTT TCC GAG CTG GAT GTT |
| | Reverse | AGG AAC CTC TCC ACT TCG CAT C |
| *Olig2* | Forward | AGA CCG AGC CAA CAC CAG |
| | Reverse | AAG CTC TCG AAT GAT CCT TCT TT |
| *Pax6* | Forward | GTT CCC TGT CCT GTG GAC TC |
| | Reverse | ACC GCC CTT GGT TAA AGT CT |
| *Sox1* | Forward | ATA CCC CCA AAA TGC ATC AA |
| | Reverse | GGA AAC GGG CTT TTC TCT CT |
| *Sox11* | Forward | CAC CAC AGC CAC AAA GCG CAA |
| | Reverse | CAC ATG GGC ACA TCC AGG TT |
| *Gapdh* | Forward | TGT TCC TAC CCC CAA TGT GT |
| | Reverse | TGT GAG GGA GAT GCT CAG TG |

**Supplementary Table 2. Mapped reads in Bru-Seq experiments**

| Library | Cell Line | Number of Reads | Number of Unique Mapped Reads |
|---|---|---|---|
| HA8mNPC0h5a | HA-8 | 72,860,158 | 44,967,039 |
| HA8mNPC0h6a | HA-8 | 62,109,913 | 41,434,129 |
| HA8mNPC0h7a | HA-8 | 51,801,745 | 32,347,723 |
| HC6mNPC0h5a | HC-6 | 62,130,545 | 56,056,777 |
| HC6mNPC0h6a | HC-6 | 64,740,891 | 58,451,291 |
| HC6mNPC0h7a | HC-6 | 55,515,348 | 49,884,359 |

* HA-8 refers to *Chd7*$^{+/+}$
HC-6 refers to *Chd7*$^{Gt/Gt}$
